# Self-production of tissue factor-coagulation factor VII complex by ovarian cancer cells

**DOI:** 10.1038/sj.bjc.6605406

**Published:** 2009-11-10

**Authors:** N Yokota, S Koizume, E Miyagi, F Hirahara, Y Nakamura, K Kikuchi, W Ruf, Y Sakuma, E Tsuchiya, Y Miyagi

**Affiliations:** 1 Molecular Pathology and Genetics Division, Kanagawa Cancer Center Research Institute, 1-1-2 Nakao Asahi-ku, 241-0815 Yokohama, Japan; 2 Department of Obstetrics, Gynecology and Molecular Reproductive Science, Yokohama City University Graduate School of Medicine, 3-9 Fukuura Kanazawa-ku, 236-0004 Yokohama, Japan; 3 Molecular Cell Biology Division, Kanagawa Cancer Center Research Institute, 1-1-2 Nakao Asahi-ku, 241-0815 Yokohama, Japan; 4 Department of Immunology and Microbial Science, The Scripps Research Institute, 10550 North Torrey Pines Road, La Jolla, CA 92037, USA

**Keywords:** coagulation factor VII, tissue factor, hypoxia, ovarian cancer, TF-positive microparticle

## Abstract

**Background:**

Thromboembolic events are a major complication in ovarian cancer patients. Tissue factor (TF) is frequently overexpressed in ovarian cancer tissue and correlates with intravascular thrombosis. TF binds to coagulation factor VII (fVII), changing it to its active form, fVIIa. This leads to activation of the extrinsic coagulation cascade. fVII is produced by the liver and believed to be supplied from blood plasma at the site of coagulation. However, we recently showed that ovarian cancer cells express fVII transcripts under normoxia and that this transcription is inducible under hypoxia. These findings led us to hypothesise that ovarian cancer cells are intrinsically associated with TF-fVIIa coagulation activity, which could result in thrombosis.

**Methods:**

In this study, we examined whether ectopically expressed fVII could cause thrombosis by means of immunohistochemistry, RT–PCR, western blotting and flow cytometry.

**Results:**

Ectopic fVII expression occurs frequently in ovarian cancers, particularly in clear cell carcinoma. We further showed that ovarian cancer cells express TF-fVIIa on the cell surface under normoxia and that this procoagulant activity is enhanced by hypoxic stimuli. Moreover, we showed that ovarian cancer cells secrete microparticles (MPs) with TF-fVIIa activity. Production of this procoagulant secretion is enhanced under hypoxia.

**Conclusion:**

These results raise the possibility that cancer cell-derived TF-fVIIa could cause thrombotic events in ovarian cancer patients.

Venous thromboembolism (VTE) is a common complication in cancer patients and a significant cause of morbidity and mortality (Chew *et al*, 2007; Varki, 2007). The risk of VTE is highest for cancers of the ovary, pancreas and liver (Iodice *et al*, 2008). In fact, ovarian cancer patients closely associate with intravascular thrombosis (Tateo *et al*, 2005; Satoh *et al*, 2007), and the development of VTE within 2 years is a strong predictor of death due to this cancer (Rodriguez *et al*, 2007). Activation of platelets and elevation of plasma tissue factor (TF) levels (Han *et al*, 2006; Tesselaar *et al*, 2007) are candidate determinants for the development of thrombosis in cancer patients.

Numerous studies have suggested that TF may have an important function in thrombosis in cancer patients. TF primarily presents on the cell surface as a transmembrane protein and functions as a cellular receptor for coagulation factor VII (fVII) circulating in the blood (Furie and Furie, 1988). By binding with TF, fVII is changed to its active form, fVIIa, and subsequently triggers the extrinsic coagulation cascade by activating factor X (Furie and Furie, 1988). Overexpression of TF correlates with a high incidence of VTE in pancreatic (Khorana *et al*, 2007) and ovarian (Uno *et al*, 2007) cancer patients. We have also reported that TF is constitutively expressed in many tumour cell lines (Koizume *et al*, 2006). Functionally active TF also circulates in the blood as a component of cell-derived microparticles (MPs) produced by platelets, cells with a monocyte/macrophage lineage (Lopez *et al*, 2005) and cancer cells (Amin *et al*, 2008). It is likely that this circulating TF may contribute to the development of thrombosis.

Hypoxia is a condition closely associated with cancer (Dewhirst *et al*, 2008), including ovarian cancer (Kim *et al*, 2006). The gene encoding TF is activated under hypoxia by a transcription factor, Egr-1, found in glioblastoma (Rong *et al*, 2005), melanoma, breast, lung (Amirkhosravi *et al*, 1998) and cervical cancer cells (Denko *et al*, 2000). To date, hypoxic induction of TF in ovarian cancer cells has not been reported.

In an earlier study, we showed that fVII, primarily synthesised in the liver, was constitutively produced in various non-hepatic cancer cells including ovarian cancer cells (Koizume *et al*, 2006). Furthermore, fVII transcription was inducible under hypoxic conditions by the hypoxia-inducible factor-2*α* (HIF-2*α*)-dependent pathway in ovarian cancer cells (Koizume *et al*, 2006). Thus, we surmised that fVII and TF induced by hypoxia in ovarian cancer cells, and not fVII derived from circulating blood, may be involved in thrombotic events in ovarian cancer patients.

The aim of this study was to examine the possibility that ovarian cancer tissue could intrinsically produce the TF-fVIIa complex independently of fVII supplied from blood plasma, and thus increase the risk of thrombosis. To this end, we investigated the expression of TF and fVII and the secretion of plasma membrane-derived MPs in ovarian cancer cells under both normoxia and hypoxia.

## Materials and methods

### Cell lines and cell culture

Ovarian cancer cell lines including four clear cell carcinoma (OVSAYO, OVISE, OVMANA, and OVTOKO); two serous adenocarcinoma (OVSAHO and OVKATE); one mucinous adenocarcinoma (MCAS); one endometrioid adenocarcinoma (TOV112D) and three undifferentiated carcinoma (KURAMOCHI, NIH:OVCAR-3, and TYK-nu) cell lines were used in this study. TOV112D and NIH:OVCAR-3 were obtained from the American Type Culture Collection (Manassas, VA, USA). Other cell lines were described earlier (Koizume *et al*, 2006). All cell lines were cultured in RPMI-1640 medium supplemented with 10% foetal bovine serum (Moregate, Brisbane, Australia) at 37°C in a humidified atmosphere under 5% CO_2_.

### Real-time RT–PCR analysis of FVII and TF gene expression

Cancer cells were harvested at the exponential growth phase. Cells (2 × 10^6^ cells) were seeded on to a 100 mm-diameter dish and cultured for 16 h. Cells were further cultured under normoxia or hypoxia (1% O_2_) as described earlier (Koizume *et al*, 2006). Total RNA isolated from cancer cells was subjected to real-time RT–PCR analysis as described earlier (Koizume *et al*, 2006). The PCR primers used for TF expression were 5′-TAACCGGAAGAGTACAGACAGC-3′ and 5′-CACTCCTGCCTTTCTACACTTG-3′. The hybridisation probes used were 5′-ATCATTGGAGCTGTGGTATTTGTGG-FITC-3′ and 5′-LCRed640-CATCATCCTTGTCATCATCCTGGC-3′. Expression of porphobilinogen deaminase was analysed as described earlier (Koizume *et al*, 2006) to normalise data.

### Isolation of MPs

To isolate cell-derived MPs, conditioned media were filtered through a 5 *μ*m-pore membrane, then ultracentrifuged at 100 000 **g** for 90 min at 10°C according to a published procedure (Davila *et al*, 2008). To isolate MPs secreted under serum-free condition, cultured cells were washed twice with 5 ml of PBS, then cultured in serum-free media for 3 h. Media were then replaced with new serum-free media and further cultured under normoxia or hypoxia for 24 h. Obtained conditioned media were processed as described above.

### fXa generation assay

The activated factor X (fXa) generation assay was performed as described earlier (Koizume *et al*, 2006) with a slight modification of seeded cell numbers (1 × 10^5^ cells per well of a 24-well plate). Briefly, conditioned media were collected and filtered through a 5 *μ*m-pore filter (Millex-SV, Millipore, Billerica, MA, USA) to remove cell debris. Six microlitres of 250 mM CaCl_2_ (final concentration was 5 mM) and 175 nM fX were added to 294 *μ*l of the filtered conditioned medium. After incubation at 37°C for 4 and 2 h for cells and conditioned media, respectively, a 20 *μ*l aliquot was removed and used for the analysis. In addition, MPs were resuspended in reaction buffer and subjected to fXa generation analysis.

### The tilt tube plasma clotting assay

Tilt tube plasma clotting assay was performed as described earlier (Rong *et al*, 2005) with slight modifications. The filtered conditioned medium (200 *μ*l) was mixed with 200 *μ*l of citrated normal human plasma (George King Bio-Medical, Inc., Overland Park, KS, USA), and then 200 *μ*l of 25 mM CaCl_2_ was added to the tube to initiate the semisolid gel formation. The times required for clot formation were recorded to evaluate procoagulant activity.

### Flow cytometry

MPs of 15 *μ*l were diluted with 85 *μ*l of binding buffer (0.01 M Hepes [pH 7.4], 140 mM NaCl and 2.5 mM CaCl_2_), and then each sample was incubated with 5 *μ*l of FITC annexin V (BD-Pharmingen, San Jose, CA, USA) and/or 20 *μ*l of PE-conjugated monoclonal anti-TF antibody (BD-Pharmingen) for 15 min at room temperature. To detect fVII on the MPs, diluted MPs were incubated with 10 *μ*l of monoclonal anti-fVII antibody (F8146, Sigma, Saint Louis, MO, USA) labelled with FITC using DyLight Antibody Labeling Kits (Thermo Scientific, Rockford, IL, USA). We used unlabelled samples, an isotype-matched mouse IgG-PE or mouse IgG-FITC (BD-Pharmingen) as controls. MPs were analysed by size, using 1 *μ*m calibration microspheres (Molecular Probes Inc., Eugene, OR, USA), and fluorescence using a Coulter Epics Altra flow cytometer (Beckman Coulter Inc., San Diego, CA, USA).

### Western blotting

Immunoblotting was performed using whole-cell lysates of cancer cells. The antibodies used were the anti-TF monoclonal antibody (TF9 10H10), the anti-Egr-1 (sc-110, Santa Cruz Biotechnology Inc., CA, USA), the anti-Sp-1 (sc-59, Santa Cruz) and the anti-*β*-actin (A5441, Sigma) antibodies. Detection of antibodies was achieved using enhanced chemiluminescence reagents (Thermo Scientific).

### Immunohistochemistry

The institutional review board at Yokohama City University approved this study. Surgically removed ovarian carcinomas were obtained from the Department of Obstetrics and Gynecology, Yokohama City University Hospital. Routinely processed formalin-fixed paraffin-embedded specimens were sectioned and stained with antibodies for TF (TF CPT at 7.6 *μ*g ml^−1^) and fVII (fVII R0882 at 10 *μ*g ml^−1^). Immunoreactivity was visualised by the peroxidase-labelled amino acid polymer method with Histofine simple stain MAX-PO(R) (Nichirei Co., Tokyo, Japan) and by the avidin–biotin–peroxidase complex method (LSAB+, DakoCytomation Co., Tokyo, Japan) according to the manufacturer's instructions. Sections were counterstained with haematoxylin. Data were obtained using the modified German immunoreactive score (Krajewska *et al*, 1996). Briefly, immunostaining intensity was rated using four ranks ranging from zero (none) to three (intense). The numbers of immunoreactive cancer cells were also estimated in four grades as zero (none), one (1–10% per cancer cells), two (10–50%) and three (50%<). Two representative areas were evaluated for each case, and the sum of the scores was used for the score of that case. Two independent examiners scored each case and the average value was used for analyses.

## Results

### Ectopic expression of fVII in human epithelial ovarian carcinoma

**Figure 1:**
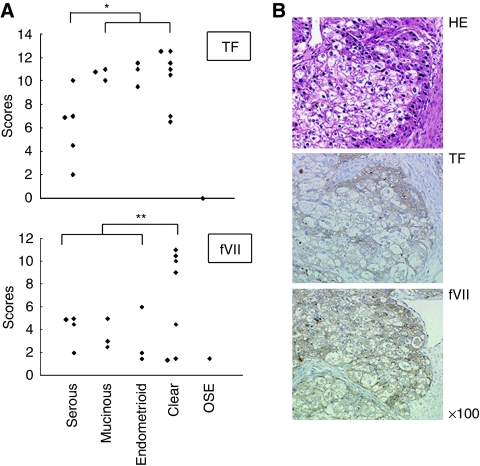
Immunohistochemical analysis of TF and fVII expressions in ovarian cancer tissues. (**A**) Expression level of TF and fVII in ovarian cancer and normal ovarian surface epithelium (OSE) of a pre-menopausal ovary. Ovarian cancer tissues from serous, mucinous, endometrioid adenocarcinomas, clear cell carcinoma and OSE were stained with TF or fVII antibody. Expression levels were rated using the modified German immunoreactive score. ^*^P=0.002, ^**^P=0.039 (Student's *t*-test). (**B**) A representative example of immunohistochemical detection of TF and fVII in an ovarian clear cell carcinoma tissue that was scored as 11.5 and 11.0 for TF and fVII, respectively.

We first examined the expression of TF and fVII in ovarian cancer tissues using 20 surgically removed specimens. The specimens consisted of five serous, three mucinous and three endometrioid adenocarcinomas, as well as seven clear cell carcinomas and one normal premenopausal ovary with normal ovarian surface epithelium (OSE) (Figure 1). Immunohistochemical analysis revealed a strong TF expression in mucinous and endometrioid adenocarcinomas, in addition to clear cell carcinomas as reported earlier (Uno *et al*, 2007) (Figure 1A and B). TF was less strongly expressed in serous adenocarcinomas. TF was not detected in OSE (Figure 1A). Immunohistochemistry also revealed that fVII was expressed in all ovarian cancer specimens examined, particularly in clear cell carcinomas (Figure 1A and B).

### Hypoxia induction of fVII and TF transcription in ovarian cancer cells of various histological types

**Figure 2:**
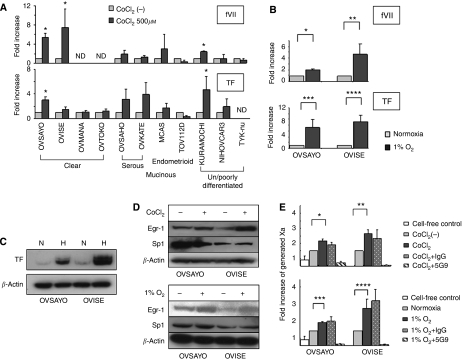
Real-time RT–PCR analysis of TF and fVII expressions in ovarian cancer cells. (**A**) Relative fVII and TF mRNA levels in ovarian cancer cells stimulated with 500 *μ*M CoCl_2_ for 4 h. fVII transcription in OVSAYO and OVSAHO cells was reported earlier (Koizume *et al*, 2006) but was re-examined for comparison. ^*^P<0.05, ND; not detected. (**B**) Relative increase of fVII and TF transcripts in OVSAYO and OVISE cells cultured under 1% O_2_ for 24 h compared with those cultured under normoxia (^*^P<0.001, ^**^P=0.023, ^***^P=0.016, ^****^P=0.003). Columns for fVII and TF mRNA levels, mean (*n*=3); Bars, s.d. Data were statistically analysed using Student's *t*-test. (**C**) Western blotting analysis of TF levels in ovarian cancer cells. N and H are indicative of normoxia and hypoxia, respectively. (**D**) Western blotting analysis of Egr-1 and Sp1 expressions in ovarian cancer cells cultured under normoxia, hypoxia (1% O_2_ for 16 h) or hypoxia mimic (500 *μ*M CoCl_2_ for 4 h) conditions. (**E**) Induction of procoagulant activity on the cell surface of ovarian cancer cells under CoCl_2_ or hypoxic conditions. Ovarian cancer cells were cultured under 500 *μ*M CoCl_2_ for 11 h or under 1% O_2_ for 16 h, and then subjected to fXa generation assay. Columns for clotting time, mean (*n*=3); Bars, s.d. ^*^P=0.001, ^**^P=0.002, ^***^P<0.001, ^****^P=0.012.

Under normoxia, we showed earlier that OVSAYO, OVISE, OVSAHO and OVTAKE cells weakly expressed fVII mRNA and that KURAMOCHI strongly expressed fVII mRNA. In the past, hypoxic FVII gene induction has been examined in detail only for OVSAYO cells (Koizume *et al*, 2006). Thus, in this study, we examined whether the FVII gene was inducible using hypoxia in additional ovarian cancer cell lines. We first used 500 *μ*M CoCl_2_ to mimic hypoxic conditions, as this approach has been shown to stimulate higher levels of HIFs in cancer cells, as compared with real hypoxic (1% O_2_) conditions (Koizume *et al*, 2008). Real-time RT–PCR analysis revealed that fVII transcription was clearly inducible by CoCl_2_ in several cell lines (Figure 2A). Constitutively expressed TF mRNA in ovarian cancer cells (Koizume *et al*, 2006) was also found to be upregulated in some cells after CoCl_2_ treatment (Figure 2A).

We next carried out a study under real hypoxic conditions (1% O_2_ for 24 h) for fVII and TF transcriptional activation in clear cell carcinoma cell lines OVSAYO and OVISE. Immunohistochemistry and RT–PCR analyses revealed that this histological subtype could highly express fVII and TF transcripts. Quantitative RT–PCR analysis revealed that real hypoxia also induced fVII and TF transcriptions (Figure 2B). TF mRNA levels were quite high under normoxia and hypoxia, compared with those of ectopically expressed fVII (Koizume *et al*, 2006), enabling us to identify TF protein by western blotting (Figure 2C).

Transcription factor Egr-1 is known to be upregulated and has a dominant function over Sp1 for TF gene induction under hypoxia (Yan *et al*, 1998; Rong *et al*, 2006). As a consequence, we additionally tested the expression of Egr-1 in ovarian cancer cell lines. Western blotting revealed that Egr-1 expression was enhanced under CoCl_2_ or 1% O_2_ conditions. In contrast, expression of Sp1, which is responsible for basal TF transcription, was unchanged in these cell lines (Figure 2D).

### Hypoxia-induced procoagulant activity in ovarian cancer cells

The TF-fVIIa complex cleaves fX to generate fXa, further activating the extrinsic coagulation cascade. To evaluate whether TF and fVII expressions in ovarian cancer cells caused the procoagulant reaction, we performed the fXa generation assay. OVSAYO and OVISE cells were cultured under normoxic, mimicked hypoxic (500 *μ*M CoCl_2_) and real hypoxic (1% O_2_) conditions. They were then tested for coagulation activity. We found that the fXa-generating activity of OVSAYO cells (10.6 pM per min) and OVISE cells (13.7 pM per min) was significantly higher than that of the cell-free control experiment (Figure 2E), suggesting that OVSAYO and OVISE cells expressed procoagulant activity on their surface under normoxia. As expected, both real hypoxic and CoCl_2_-induced conditions enhanced procoagulant activity on OVSAYO and OVISE cells (Figure 2E). Further, the addition of anti-TF (5G9) antibody markedly reduced the procoagulant activity, although normal IgG failed to inhibit it (Figure 2E). This suggested that the TF-fVIIa complex was responsible for the observed procoagulant activity.

### TF-fVIIa activity in ovarian cancer cells under normoxia and hypoxia

**Figure 3:**
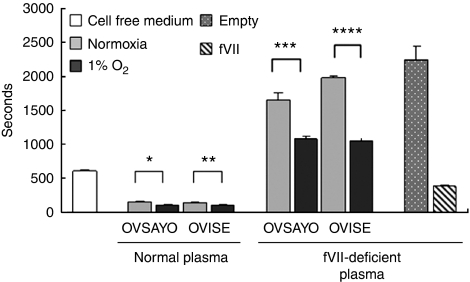
Secretion of procoagulant activity by ovarian cancer cells. Ovarian cancer cells were cultured under normoxia or 1% O_2_ for 24 h, and then conditioned media were subjected to the plasma clotting assay using normal or fVII-deficient human plasma. FVII-containing cells and empty cells were indicative of stably fVII cDNA-transfected cells and stably transfected empty vector cells, respectively. Columns for clotting time, mean (*n*=3); Bars, s.d. ^*^P=0.009, ^**^P=0.003, ^***^P=0.002, ^****^P=0.0001.

Increased plasma TF levels have been reported to correlate with thrombosis in several cancer patients. In this context, we showed that ovarian cancer cells could intrinsically express TF-fVIIa activity on their surface. Thus, we next investigated whether ovarian cancer cells could secrete the TF-fVIIa complex without fVII derived from blood plasma. OVSAYO and OVISE cells were cultured under normoxia or 1% O_2_, and conditioned media were subsequently collected. These conditioned media were then mixed with citrated human normal plasma supplemented with CaCl_2_, and gently rocked for the tilt tube assay. The clotting time achieved using conditioned media, prepared from cells cultured under normoxia, was markedly reduced as compared with experiments using cell-free medium (Figure 3). As expected, experiments with conditioned media, prepared from cells cultured under hypoxia, revealed that clotting times were further shortened (Figure 3). The coagulation reactions were completely blocked by 5G9 antibody treatment (data not shown). These results suggested that ovarian cancer cells could secrete functional TF.

We further performed the same clotting assay using fVII-deficient plasma. As expected, clotting times were generally longer than those recorded in experiments using normal fVII-containing plasma (Figure 3). However, plasma clotting was considerably enhanced under hypoxia in the absence of fVII (Figure 3). As expected, positive control experiments using fVII-overexpressing OVSAYO cells (Koizume *et al*, 2006) showed further reduced clotting times, in comparison with experiments involving empty vector-transfected cells (Figure 3). This suggested that ectopically synthesised fVII is secreted as a functional procoagulant in ovarian cancer cells.

### Secretion of MPs with TF-fVIIa activity by ovarian cancer cells

**Figure 4:**
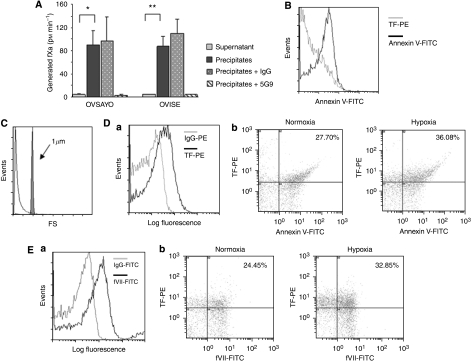
Secretion of MPs with TF-fVIIa activity by ovarian cancer cells. (**A**) fXa generation activity of the supernatant and precipitate fraction of conditioned media prepared from OVSAYO and OVISE cells cultured under 1% O_2_. Procoagulant activity predominantly presented in the precipitate fraction and this activity was completely blocked by anti-TF (5G9) antibody treatment. ^*^P<0.001, ^**^P=0.001. (**B**) Flow cytometry analysis of precipitate fraction immunostained with PE-coupled anti-TF antibody and/or FITC-coupled annexin V showed that precipitate fraction prepared from conditioned media of OVSAYO cells was annexin-V positive. (**C**) The extracellular procoagulants analysed by flow cytometry. The grey peak corresponds to the 1-*μ*m calibration microspheres. The white peak corresponds to the precipitate fraction prepared from conditioned media of OVSAYO cells. (**D**) Flow cytometry analysis of the precipitate fractions prepared from serum-free conditioned media of OVSAYO cells cultured under normoxia or 1% O_2_ for 24 h. (**Da**) The MP fraction prepared was TF positive. Background fluorescence was set with a fluorescent mouse IgG control. (**Db**) Dot plot of MP fraction immunostained with PE-coupled anti-TF antibody and FITC-coupled annexin V. Percentages shown in the upper right segment of the figure denote the relative amount of TF-positive MPs. (**E**) Flow cytometry analysis of the precipitate fractions prepared from serum-free conditioned media of OVSAYO cells cultured under normoxia or 1% O_2_ for 24 h. (**Ea**) The MP fraction prepared was fVII positive. Background fluorescence was set with a fluorescent mouse IgG control. (**Eb**) Dot plot of MP fraction immunostained with PE-coupled anti-TF antibody and FITC-coupled anti-fVII antibody. Percentages in the upper right segment of the figure denote amounts of fVII-positive fractions relative to total MPs.

TF has been shown to circulate in the blood as a component of cell-derived MPs released as fragments of plasma membrane. Secretion of TF-positive MPs has been found to be increased and associated with the activation of the haemostatic system in several cancer patients (Hron *et al*, 2007; Davila *et al*, 2008). Following up on this finding, we next investigated whether TF-fVIIa secreted from ovarian cancer cells was associated with the activity of MPs. Conditioned media from ovarian cancer cell culture under 1% O_2_ were ultracentrifuged. Coagulation activity was then assessed for both supernatant and precipitate fractions. The fXa generation assay revealed that TF-fVIIa activity was predominantly enhanced in the precipitate that included MPs (Figure 4A). As expected, procoagulant activity was blocked by anti-human TF antibody treatment (Figure 4A).

We performed flow cytometry to further examine whether the activity of TF-fVIIa secreted by ovarian cancer cells was associated with MPs. The analysis revealed that most of the particles (∼90%) present in the precipitate from the serum-free conditioned media bind to annexin V. They were smaller than 1 *μ*m in size (Figure 4B and C), thus satisfying the criteria for being MPs. MPs were also found to be TF positive by FACS analysis (Figure 4Da). The number of TF-positive MPs secreted into serum-free conditioned media from OVSAYO cells, maintained under 1% O_2,_ increased by ∼1.3 times compared with total MPs, including microvesicles not associated with TF (Figure 4Db). Flow cytometry with anti-human fVII antibody showed that the TF-positive MP fraction prepared from serum-free culture media was also fVII positive (Figure 4Ea and Eb), eliminating the possibility that pre-existing trace fVII in bovine serum was responsible for binding TF-positive MPs. The number of fVII-positive MPs was increased under hypoxia relative to the number of TF-positive MPs (Figure 4E). These results suggested that ovarian cancer cells secreted TF-fVIIa-positive MPs.

## Discussion

In this study, we showed that not only TF but also fVII proteins were localised to cancer cells in surgically removed ovarian cancer tissue specimens. This was especially true in the case of clear cell carcinomas. Unlike TF expressions, the fVII expression evaluated by immunohistochemistry was generally weak, except for clear cell carcinomas, and was comparable with that of a normal ovarian tissue. fVII that came from blood plasma perhaps cross-reacts in histochemical analysis. Furthermore, we found that hypoxic conditions induced the expression of the FVII gene, together with the TF gene, in various types of ovarian cancer cells. We showed that slight TF-fVIIa activity on the surface of ovarian cancer cells under normoxia was enhanced under hypoxic conditions. We further showed that TF-fVIIa activity was not only localised on the cancer cell surface, but was also secreted into culture media as a component of MPs. Hypoxia was found to accelerate this process.

The TF-fVIIa complex on the ovarian cancer cell surface may trigger the extrinsic coagulation cascade within or around ovarian cancer tissue. TF-fVIIa on the cell surface is also known to transduce various kinds of signals within cells. Both the products of the coagulation cascade and transduced signals in cells have important functions in cancer biology, such as cell invasion and migration, angiogenesis and cell survival through intracellular signalling mechanisms (Ruf, 2007). We showed earlier that ectopic synthesis of fVII in OVSAYO cells, followed by TF-fVIIa formation on the cell surface, markedly enhanced cell motility and invasion (Koizume *et al*, 2006). Ovarian cancer is highly metastatic and tumour cell migration from the primary site to the peritoneal cavity is the critical process of ovarian cancer spread. Given that cells in primary ovarian cancer tissue and ovarian cancer cells in ascites undergo hypoxic stress (Kim *et al*, 2006), self-production of TF-fVIIa on the cell surface may contribute to metastasis of ovarian cancer.

In addition to cell surface localisation, TF could circulate in the blood even under normal conditions. TF is found in the blood as an alternatively spliced soluble form without the transmembrane domain or as a component of cell-derived MPs. Most of the TF-derived procoagulant activity is associated with MPs (Yu and Rak, 2004). TF-positive MPs in different types of cancer patients are known to increase in response to various cellular stresses (Amin *et al*, 2008). It is likely that cancer cell-derived MPs contribute to hypercoagulability in those patients, as various types of cancer cells are known to secrete TF-positive MPs (Amin *et al*, 2008). In these cases, procoagulant activity of TF-positive MPs was considered to depend on blood-derived fVII. In this study, we have provided novel evidence that MPs containing active TF-fVIIa complex are secreted directly from ovarian cancer cells, and that this phenomenon is enhanced under hypoxia. Given that ovarian cancer patients have a high incidence of VTE at distant sites from neoplastic tissue (Tateo *et al*, 2005; Satoh *et al*, 2007), cancer cell-derived TF-fVIIa-positive MPs may have a function in intravascular thrombosis in such patients.

Elevated incidences of VTE have been reported to be associated with tumour size in gliomas (Marras *et al*, 2000). Indeed, increase in tumour volume is associated with progressive development of hypoxia in many cancer tissues. Thus, it is probable that hypoxic stresses that are promoted as ovarian cancer grows, especially in the peritoneal cavity, accentuate the secretion of TF-fVIIa-positive MPs, leading to the development of VTE. Future investigations into the relationship between the ectopic expression of fVII in tumour tissue; plasma concentrations of TF or TF-fVIIa-positive MPs; and the incidence of VTE in ovarian cancer patients will lead to a greater understanding of the mechanisms of thrombosis in cancer patients.

## References

[bib1] Amin C, Mackman N, Key NS (2008) Microparticles and cancer. Pathophysiol Haemost Thromb 36: 177–183 19176990 10.1159/000175155

[bib2] Amirkhosravi A, Meyer T, Warnes G, Amaya M, Malik Z, Biggerstaff JP, Siddiqui FA, Sherman P, Francis JL (1998) Pentoxifylline inhibits hypoxia-induced upregulation of tumor cell tissue factor and vascular endothelial growth factor. Thromb Haemost 80: 598–602 9798977

[bib3] Chew HK, Wun T, Harvey DJ, Zhou H, White RH (2007) Incidence of venous thromboembolism and the impact on survival in breast cancer patients. J Clin Oncol 25: 70–76 17194906 10.1200/JCO.2006.07.4393

[bib4] Davila M, Amirkhosravi A, Coll E, Desai H, Robles L, Colon J, Baker CH, Francis JL (2008) Tissue factor-bearing microparticles derived from tumor cells: impact on coagulation activation. J Thromb Haemost 6: 1517–1524 18433463 10.1111/j.1538-7836.2008.02987.x

[bib5] Denko N, Schindler C, Koong A, Laderoute K, Green C, Giaccia A (2000) Epigenetic regulation of gene expression in cervical cancer cells by the tumor microenvironment. Clin Cancer Res 6: 480–487 10690527

[bib6] Dewhirst MW, Cao Y, Moeller B (2008) Cycling hypoxia and free radicals regulate angiogenesis and radiotherapy response. Nat Rev Cancer 8: 425–437 18500244 10.1038/nrc2397 PMC3943205

[bib7] Furie B, Furie BC (1988) The molecular basis of blood coagulation. Cell 53: 505–518 3286010 10.1016/0092-8674(88)90567-3

[bib8] Han LY, Landen Jr CN, Kamat AA, Lopez A, Bender DP, Mueller P, Schmandt R, Gershenson DM, Sood AK (2006) Preoperative serum tissue factor levels are an independent prognostic factor in patients with ovarian carcinoma. J Clin Oncol 24: 755–761 16380413 10.1200/JCO.2005.02.9181

[bib9] Hron G, Kollars M, Weber H, Sagaster V, Quehenberger P, Eichinger S, Kyrle PA, Weltermann A (2007) Tissue factor-positive microparticles: cellular origin and association with coagulation activation in patients with colorectal cancer. Thromb Haemost 97: 119–123 17200778

[bib10] Iodice S, Gandini S, Lohr M, Lowenfels AB, Maisonneuve P (2008) Venous thromboembolic events and organ-specific occult cancers: a review and meta-analysis. J Thromb Haemost 6: 781–788 18284604 10.1111/j.1538-7836.2008.02928.x

[bib11] Khorana AA, Ahrendt SA, Ryan CK, Francis CW, Hruban RH, Hu YC, Hostetter G, Harvey J, Taubman MB (2007) Tissue factor expression, angiogenesis, and thrombosis in pancreatic cancer. Clin Cancer Res 13: 2870–2875 17504985 10.1158/1078-0432.CCR-06-2351

[bib12] Kim KS, Sengupta S, Berk M, Kwak YG, Escobar PF, Belinson J, Mok SC, Xu Y (2006) Hypoxia enhances lysophosphatidic acid responsiveness in ovarian cancer cells and lysophosphatidic acid induces ovarian tumor metastasis *in vivo*. Cancer Res 66: 7983–7990 16912173 10.1158/0008-5472.CAN-05-4381

[bib13] Koizume S, Jin MS, Miyagi E, Hirahara F, Nakamura Y, Piao JH, Asai A, Yoshida A, Tsuchiya E, Ruf W, Miyagi Y (2006) Activation of cancer cell migration and invasion by ectopic synthesis of coagulation factor VII. Cancer Res 66: 9453–9460 17018600 10.1158/0008-5472.CAN-06-1803

[bib14] Koizume S, Yokota N, Miyagi E, Hirahara F, Tsuchiya E, Miyagi Y (2008) Heterogeneity in binding and gene-expression regulation by HIF-2alpha. Biochem Biophys Res Commun 371: 251–255 18423372 10.1016/j.bbrc.2008.04.042

[bib15] Krajewska M, Krajewski S, Epstein JI, Shabaik A, Sauvageot J, Song K, Kitada S, Reed JC (1996) Immunohistochemical analysis of bcl-2, bax, bcl-X, and mcl-1 expression in prostate cancers. Am J Pathol 148: 1567–1576 8623925 PMC1861561

[bib16] Lopez JA, del Conde I, Shrimpton CN (2005) Receptors, rafts, and microvesicles in thrombosis and inflammation. J Thromb Haemost 3: 1737–1744 16102040 10.1111/j.1538-7836.2005.01463.x

[bib17] Marras LC, Geerts WH, Perry JR (2000) The risk of venous thromboembolism is increased throughout the course of malignant glioma: an evidence-based review. Cancer 89: 640–664 10931464 10.1002/1097-0142(20000801)89:3<640::aid-cncr20>3.0.co;2-e

[bib18] Rodriguez AO, Wun T, Chew H, Zhou H, Harvey D, White RH (2007) Venous thromboembolism in ovarian cancer. Gynecol Oncol 105: 784–790 17408726 10.1016/j.ygyno.2007.02.024

[bib19] Rong Y, Hu F, Huang R, Mackman N, Horowitz JM, Jensen RL, Durden DL, Van Meir EG, Brat DJ (2006) Early growth response gene-1 regulates hypoxia-induced expression of tissue factor in glioblastoma multiforme through hypoxia-inducible factor-1-independent mechanisms. Cancer Res 66: 7067–7074 16849552 10.1158/0008-5472.CAN-06-0346 PMC2610484

[bib20] Rong Y, Post DE, Pieper RO, Durden DL, Van Meir EG, Brat DJ (2005) PTEN and hypoxia regulate tissue factor expression and plasma coagulation by glioblastoma. Cancer Res 65: 1406–1413 15735028 10.1158/0008-5472.CAN-04-3376

[bib21] Ruf W (2007) Tissue factor and PAR signaling in tumor progression. Thromb Res 120(Suppl 2): S7–S12 18023716 10.1016/S0049-3848(07)70125-1

[bib22] Satoh T, Oki A, Uno K, Sakurai M, Ochi H, Okada S, Minami R, Matsumoto K, Tanaka YO, Tsunoda H, Homma S, Yoshikawa H (2007) High incidence of silent venous thromboembolism before treatment in ovarian cancer. Br J Cancer 97: 1053–1057 17895896 10.1038/sj.bjc.6603989 PMC2360447

[bib23] Tateo S, Mereu L, Salamano S, Klersy C, Barone M, Spyropoulos AC, Piovella F (2005) Ovarian cancer and venous thromboembolic risk. Gynecol Oncol 99: 119–125 15990161 10.1016/j.ygyno.2005.05.009

[bib24] Tesselaar ME, Romijn FP, Van Der Linden IK, Prins FA, Bertina RM, Osanto S (2007) Microparticle-associated tissue factor activity: a link between cancer and thrombosis? J Thromb Haemost 5: 520–527 17166244 10.1111/j.1538-7836.2007.02369.x

[bib25] Uno K, Homma S, Satoh T, Nakanishi K, Abe D, Matsumoto K, Oki A, Tsunoda H, Yamaguchi I, Nagasawa T, Yoshikawa H, Aonuma K (2007) Tissue factor expression as a possible determinant of thromboembolism in ovarian cancer. Br J Cancer 96: 290–295 17211468 10.1038/sj.bjc.6603552 PMC2359996

[bib26] Varki A (2007) Trousseau's syndrome: multiple definitions and multiple mechanisms. Blood 110: 1723–1729 17496204 10.1182/blood-2006-10-053736 PMC1976377

[bib27] Yan SF, Zou YS, Gao Y, Zhai C, Mackman N, Lee SL, Milbrandt J, Pinsky D, Kisiel W, Stern D (1998) Tissue factor transcription driven by Egr-1 is a critical mechanism of murine pulmonary fibrin deposition in hypoxia. Proc Natl Acad Sci USA 95: 8298–8303 9653181 10.1073/pnas.95.14.8298 PMC20970

[bib28] Yu JL, Rak JW (2004) Shedding of tissue factor (TF)-containing microparticles rather than alternatively spliced TF is the main source of TF activity released from human cancer cells. J Thromb Haemost 2: 2065–2067 15550054 10.1111/j.1538-7836.2004.00972.x

